# The Weather

**Published:** 1841-08

**Authors:** 


### THE WEATHER.

The range of the thermometer, this summer, has been high. In June, for many days, it rose regularly above 85°, and not unfrequently exceeded 90°. Once or twice it was seen as high as 93°. In Rutherford county, Tennessee, cholera infantum made its appearance during the first fortnight of this hot weather, and a number of cases have been so bad as to demand the attention of a physician; but a majority yielded to the domestic remedies prescribed by the parents, or to an occasional dose of calomel. In a severe case, which we saw, the most signal and immediate relief was derived from allowing the little patient to eat freely of ice. The incessant retching with which it had been tormented ceased in a few minutes after it had begun to swallow the ice. It is now perfectly restored. In another case, we thought the stage of convalescence was hastened and confirmed by the use of the prussiate of iron. Y.

